# Cassava mosaic disease in Burkina Faso: epidemiological aspects and disease management perspectives

**DOI:** 10.1007/s44279-025-00386-2

**Published:** 2025-10-07

**Authors:** Seydou Sawadogo, Fidèle Tiendrébéogo, Ezechiel B. Tibiri, Pakyendou E. Name, Florencia Djigma, Lassina Traoré, Justin S. Pita, Angela O. Eni

**Affiliations:** 1 Laboratoire de Virologie et de Biotechnologies Végétales, Institut de l’Environnement et de Recherches Agricoles (INERA), Ouagadougou, Burkina Faso; 2 Laboratoire de Biologie Moléculaire et de Génétique (LABIOGENE), Université Joseph Ki-Zerbo, Ouagadougou, Burkina Faso; 3 Pôle scientifique et d’innovation de Bingerville, Central and West African Virus Epidemiology (WAVE), Université Félix Houphouët-Boigny (UFHB), Bingerville, Côte d’Ivoire

**Keywords:** Cassava mosaic begomoviruses, Classical surveillance, Surveillance limitations, Incidence, Burkina faso

## Abstract

Cassava Mosaic Disease (CMD) is the main biotic constraint to cassava production in West Africa. In Burkina Faso, efforts are underway to manage it sustainably using classical surveillance, including field monitoring and laboratory diagnosis. However, this method has significant limitations that make it difficult to address early management. The aim of this study was to provide recent epidemiological data on CMD, while highlighting the limitations of classical surveillance. Fields monitoring and laboratory diagnosis took 14 months for the 2020 survey and 6 months for the 2022 survey year. A total of 4,680 plants from 156 fields were assessed in 2020 and 3,810 plants from 127 fields in 2022. The overall mean incidences were 9.46% and 3.33% in 2020 and 2022, respectively. The highest incidences were found in the Centre-Ouest region (23.13%) in 2020 and in the Plateau-Central region (32.38%) in 2022. The Plateau-Central region was identified as a CMD new outbreak. In 2020 survey, *African cassava mosaic virus* (ACMV) rate was 93.95%, while *East African cassava mosaic virus* (EACMV) was 3.69%. In the 2022 survey, ACMV alone was detected in 75.95%, in double infection with EACMV in 16.46%, and triple infection with EACMV and *East African cassava mosaic Cameroon virus* (EACMCMV) in 5.06%. This study showed that classical surveillance is a robust method, but extremely time consuming to anticipate early management of CMD. Similarly, classical surveillance required significant financial resources for its implementation. Given these constraints, the implementation of participatory surveillance involving farmers, extension agents, breeders, plant protection services, and researchers could help in CMD management efforts.

**Supplementary Information:**

The online version contains supplementary material available at 10.1007/s44279-025-00386-2.

## Introduction

Cassava (*Manihot esculenta* Crantz) is a staple food for about 800 million people in Africa [1]. In 2021, its global production was estimated at 315 million tonnes, with more than 203 million tonnes produced in Africa [2]. Cassava is known as an efficient crop for achieving food security, especially for low-income countries. It is resilient to harsh climatic conditions, contributes efficiently to food security and provides substantial income to smallholder farmers when production levels are satisfactory [3]. In Burkina Faso, cassava production for economic purposes is relatively new [4]. Cassava became a cash crop for smallholder farmers and contributes to the empowerment of women through its transformation into attiéké [4]. Its production in 2021 was estimated to 16,000 tonnes [2]. However, its production is threatened by cassava mosaic disease (CMD), which negatively affects yields [5–8].

CMD is the major biotic constraint to cassava production in sub-Sahara Africa. Yield losses associated with CMD range from US$1.9 to US$2.7 billion in Africa [9]. It is a viral disease caused by the B*egomovirus* genus, which belongs to the *Geminiviridae* family. Several species of begomovirus, also named Cassava Mosaic Begomoviruses (CMB), are known to infect cassava throughout the world, with the most number reported in Africa [3]. In Burkina Faso, the cassava begomovirus circulating include *African cassava mosaic virus* (ACMV), *African cassava mosaic Burkina Faso virus* (ACMBFV) and *East African cassava mosaic Cameroon virus* (EACMCMV) [8]. Symptoms caused by CMB can range from a mosaic pattern on cassava leaves to very severe symptoms, depending on the severity of the disease [10]. CMB is transmitted by the whitefly (*Bemisia tabaci*) and spread through infected cuttings used as planting material [11]. Several methods were developed for CMD and its vector effective management [11]. One of these methods is based on surveillance throughout cassava production area for disease assessment in fields.

Classical surveillance, which includes fields monitoring, laboratory diagnosis and communication of results to policy-makers, is the most widely used method for plant disease management as well as cassava virus diseases [10, 11]. This method highlights the dynamics of CMD due to its robustness and precision for epidemiological data collection. However, classical surveillance faces significant constraints that make it difficult to address early management. These limitations include the high costs associated with field and laboratory activities, as well as the time required to perform them [12, 13]. However, in developing countries, the limited availability of resources such as financial support, qualified personnel, laboratories, infrastructure and technologies makes it difficult to use classical surveillance methods to manage plant viral diseases effectively and promptly. The aim of this study was to provide recent epidemiological data on CMD in Burkina Faso, while highlighting the limitations of classical surveillance methods and exploring cost-effective approaches to managing plant viruses.

## Materials and methods

### Field survey and sampling

**Fig. 1:**
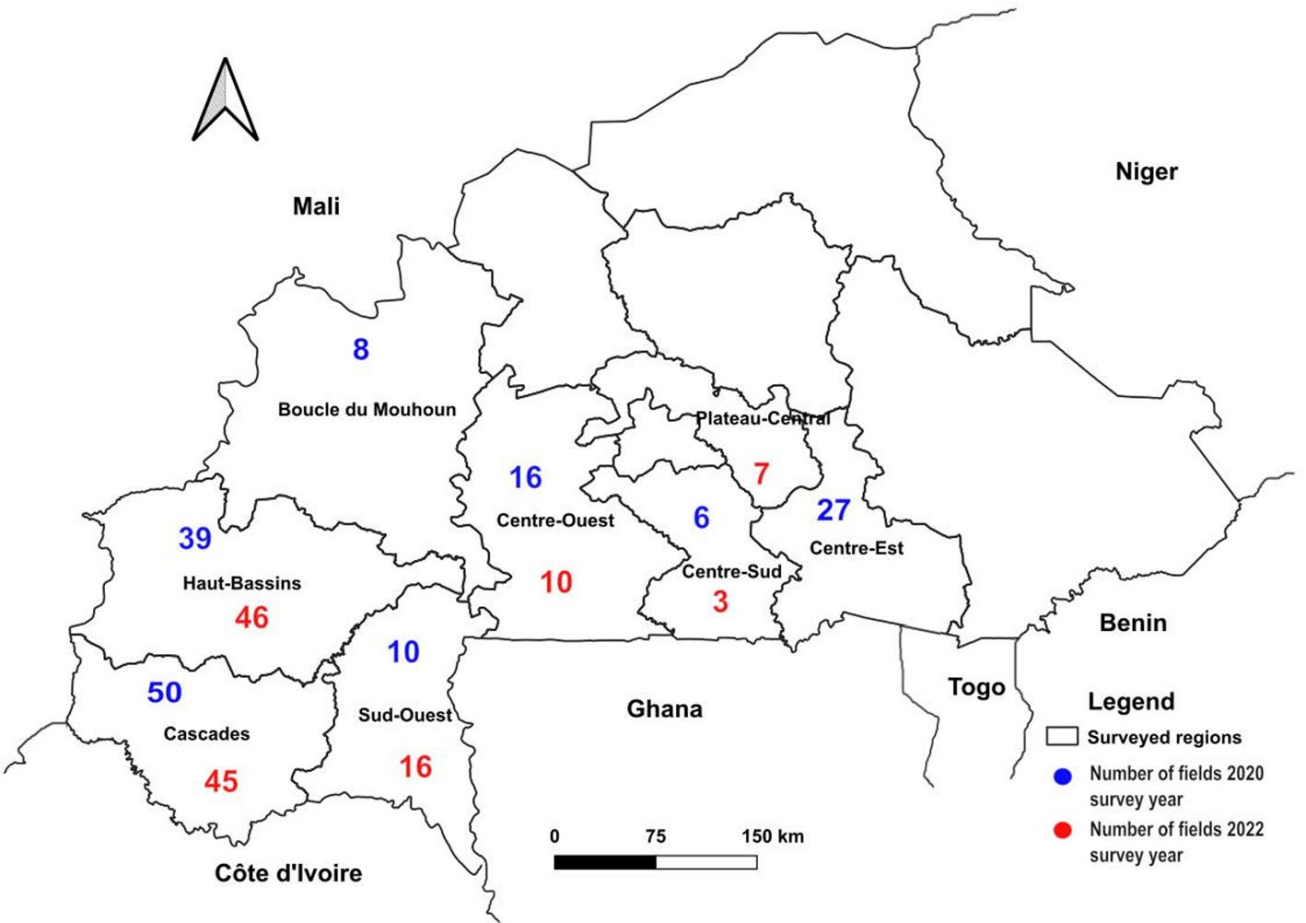
Map of Burkina Faso showing regions and number of fields surveyed in 2020 and 2022

The surveys were carried out in 2020 and 2022. Seven regions, including Boucle du Mouhoun, Cascades, Centre-Est, Centre-Ouest, Centre-Sud, Hauts-Bassins, and Sud-Ouest (2020 survey), and six regions, including Cascades, Centre-Ouest, Centre-Sud, Hauts-Bassins, Plateau-Central and Sud-Ouest (2022 survey), were surveyed (Fig. 1). The CMD assessment in cassava fields was carried out using the harmonised protocol of the Central and West African Virus Epidemiology Programme (WAVE) [10]. A road map was used to survey cassava production areas, with a distance of 10 km between sampling sites if applicable. Geo-referenced coordinates of each field were recorded using a Global Positioning System (Garmin GPS map 62s, Taiwan). The maps of CMB species distribution were generated from these coordinates using QGIS 2. 18v software. In each field, 30 cassava plants were randomly assessed visually along two diagonals in an ‘X’ form for CMD symptoms identification such as mosaic pattern, vein-clearing and leave distortion [10]. The epidemiological parameters assessed were CMD incidence, symptom severity, and whitefly abundance. Disease incidence was calculated per field, and corresponded to the number of assessed plants showing CMD symptoms out of the total number of assessed plants. CMD severity was scored from score 1 to 5 according to the observed symptoms as described [10]. Thus, the severity score of CMD was obtained by considering the sum of the severity score of infected plants in each field. The sources of CMD infection were determined according to the location of the symptoms on the infected leaves. When symptoms appeared only on the upper leaves, the infection was attributed to transmission by whiteflies. However, when both upper and lower leaves showed CMD symptoms, the infection was attributed to cutting. Whitefly abundance was estimated by counting the number of whiteflies on the five top leaves of the assessed plants. The whiteflies were then trapped and stored in 2 mL Eppendorf tubes containing 96% alcohol. Leaf samples were collected from the surveyed plants, placed in envelopes and oven-dried at 37 °C. The samples were then subjected to polymerase chain reaction (PCR) detection.

### DNA extraction and polymerase chain reactions (PCRs)

Total DNA was extracted from cassava dried leaves using the CTAB method [14]. The concentration of DNA in each sample was checked using a NanoDrop 2000 spectrophotometer (Thermo Fisher Scientific) and adjusted to approximately 100 ng/µL before PCR amplification.

PCR was performed using the WAVE primer set (Table 1) to detect ACMV, EACMV, and EACMCMV. Each reaction mixture contained PCR reaction buffer (10X), 10 mM dNTPs (Promega), 25 mM MgCl_2_, 20 pmol of each primer at 10 mM, and 1 U Taq DNA Polymerase (Promega). The PCR programme consisted of an initial denaturation step at 94 °C for 5 min, followed by 35 cycles of denaturation at 94 °C for 45 s, hybridization for 45 s, 55 °C for JSP001/JSP002 and ACMVBF/ACMVBR, 58 °C for JSP001/JSP003, 56 °C CMBRepF/EACMVRepR, 52 °C for VNF031F/VNF032R, and 70 °C for 1 min. The extension step was performed at 72 °C for 60 s, followed by a final extension at 72 °C for 10 min. The PCR products were separated by electrophoresis on a 1% agarose gel stained with ethidium bromide (10 mg/mL) using the 100-base-pair DNA ladder (Solis Biodyne, Estonia). The gel was visualised under UV light using a Compact Digimage system, series UVDI (MS Major Science).

**Table 1:** WAVE primer set used for the detection of cassava mosaic begomoviruses

Primer name	Sequence (5’ − 3’)	Target region	Size	References
sJSP 001	ATGTCGAAGCGACCAGGAGAT	ACMV DNA-A (CP)	783 bp	[17]
JSP 002	TGTTTATTAATTGCCAATACT			
ACMVBF	TCGGGAGTGATACATGCGAAGGC	ACMV DNA-B (BV1/BC1)	628 bp	[16]
ACMVBR	GGCTACACCAGCTACCTGAAGCT			
JSP 001	ATGTCGAAGCGACCAGGAGAT	EACMV DNA-A (CP)	780 bp	[17]
JSP 003	CCTTTATTAATTTGTCACTGC			
CMBRepF	CRTCAATGACGTTGTACCA	EACMV DNA-A (AC1)	650 bp	[18]
EACMVRepR	GGTTTGCAGAGAACTACATC			
VNF031F	GGATACAGATAGGGTTCCCAC	EACMV-CM DNA-A (AC2/AC3)	≈ 560 bp	[15]
VNF032R	GACGAGGACAAGAATTCCAAT			
ACMV21F	GCAGTGATGAGTTCCCCGGTGCG	DNA-A (AC3-AC2-AC1) of ACMV, EACMV	552 bp	[16]
ACMV21R	ATTCCGCTGCGCGGCCATGGAGACC			
WAVE-AB177F	GATCTGCGGGCCTATCGAAT	ACMV/BV1	800 pb	[19]
WAVE-AB977R	TTCACGCTGTGCAATACCCT			

### Data treatment and statistical analysis

The field data collected was stored in the WAVE Cube database. Data were organised for each sample of leaves and whiteflies, at the plant, field, district, and regional levels. These data were then uploaded for statistical analysis using R software version 4.0.0 [20]. Incidence, severity, and whitefly population data were analysed using analysis of variance (ANOVA). The Student-Newman-Keuls (SNK) test was used to compare means at the 5% significance level.

## Results

### Assessment of CMD symptoms, incidence, severity and whitefly abundance

**Fig. 2:**
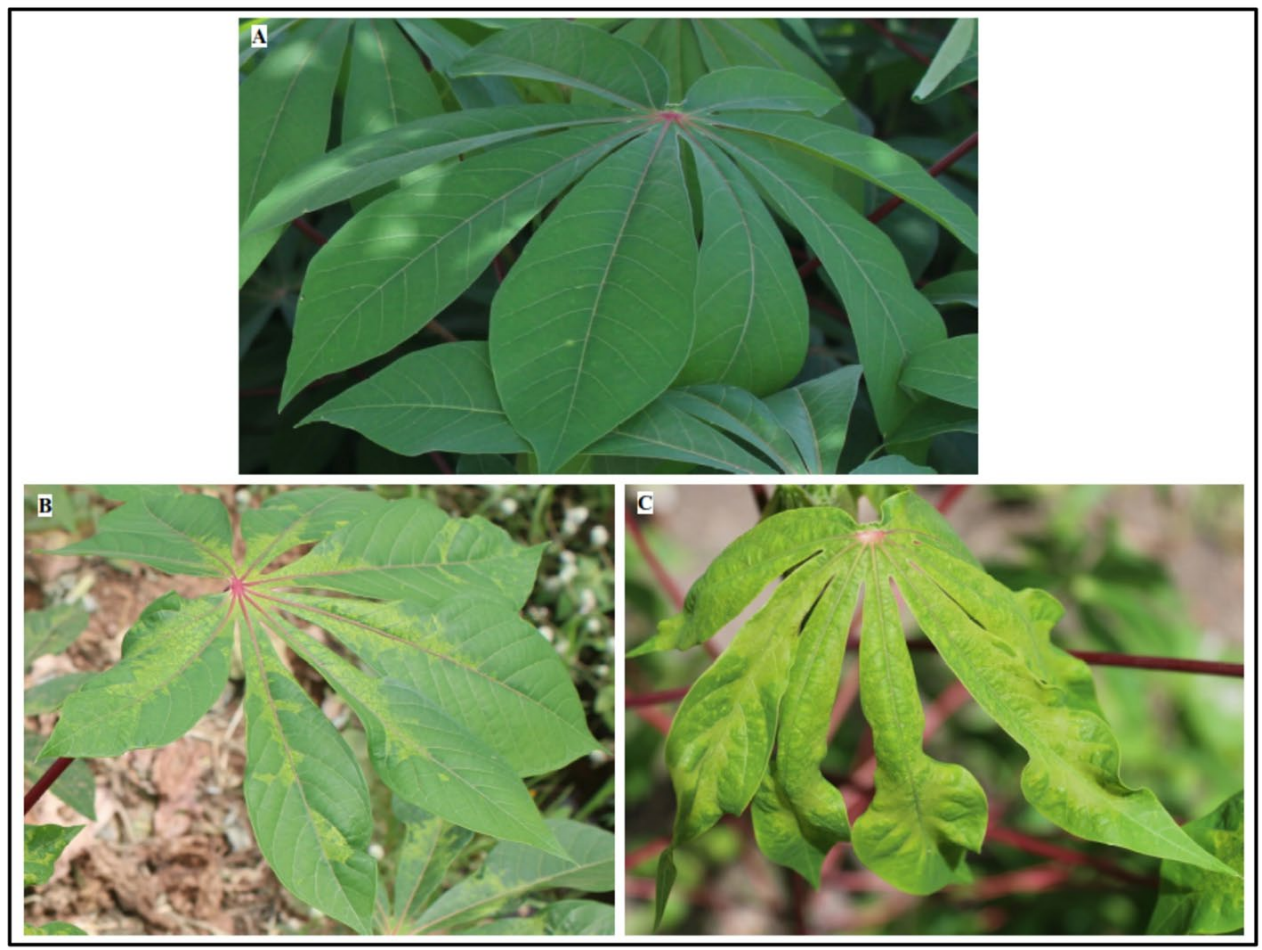
Overview of cassava leaves observed in the cassava surveyed fields. **A**: Asymptomatic leaf. **B**: Mosaic with vein-clearing. **C**: Mosaic with leaf distortion

The field survey was conducted over a period of ten months in 2020 and four months in 2022. CMD symptoms were observed in all surveyed regions except Hauts-Bassins, where no symptoms were found in 2022. In both years of the survey, the most commonly observed symptom in CMD-infected fields was a mosaic pattern accompanied by vein clearing and leaf distortion (Fig. 2). A total of 4,680 plants from 156 fields were assessed in 2020, while 3,810 plants from 127 fields were assessed in 2022. There was a statistically significant difference (*P* < 0.001) in the incidence, severity, and abundance of whiteflies between the survey years and regions (Table 2).

The overall mean incidence across all surveyed regions was 9.46% in 2020 and 3.33% in 2022. In 2020, the highest incidence was observed in the Centre-Ouest region (23.13%), while the lowest incidence was observed in Hauts-Bassins (2.31%) and Sud-Ouest (1.33%). No significant differences were observed between the Boucle du Mouhoun, Cascades, Centre-Est and Centre-Sud regions, where incidence rates ranged from 8.73% to 14.81%. In 2022, the highest incidence was observed in the Plateau-Central region (32.38%). However, the incidence remained relatively low in the Cascades, Centre-Ouest, Centre-Sud, Hauts-Bassins and Sud-Ouest regions (Table 2).

In terms of CMD severity, the overall mean scores were 2.04 in 2020 and 2.02 in 2022. Only one field in the Sud-Ouest region obtained a severity score of 3 in the 2020 survey (Table 2).

Globally, the mean number of whiteflies per assessed plant was low in all survey regions in both survey years. However, the highest number of whiteflies per assessed plant was recorded in Sud-Ouest (6.27) and Centre-Sud (4.86) in 2020 and 2022, respectively (Table 2).

**Table 2:** CMD incidence, score severity and whitefly abundance in all survey regions in 2020 and 2022

Year 2020				Year 2022			
Regions	Mean incidence (%)	Mean score Severity	Mean of whitefly abundance	Regions	Mean incidence (%)	Mean score Severity	Mean of whitefly abundance
Boucle du Mouhoun	11.25ab	2.00 b	0.43b	Cascades	2.29ab	2.03a	0.01c
Cascades	8.73ab	2.06 b	0.77b	Centre-Ouest	0.66c	2.00a	0.00c
Centre-Est	14.81ab	2.01 b	0.06b	Centre-Sud	3.33ab	2.00a	4.86a
Centre-Ouest	23.13a	2.01 b	0.49b	Hauts- Bassins	0c	1b	0.06c
Centre-Sud	12.78ab	2.00 b	0.02b	Plateau-Central	32.38a	2.07a	1.38b
Hauts- Bassins	2.31b	2.14 b	2.41b	Sud-Ouest	4.79b	2.00a	0.04c
Sud-Ouest	1.33b	3.00 a	6.27a	Overall mean	3.33	2.02	1.34
Overall mean	9.46	2.04	1.33	p-value	< 0.001	< 0.001	< 0.001
p-value	< 0.001	< 0.001	< 0.001	Significant level	***	***	***
Significant level	***	***	***				

Within the same column, values assigned the same letter are not significantly different according to the Student-Newman-Keuls (SNK) test at the 5% probality level. Values assigned different letters defer highly significantly (***) at *p* < 0.001

### CMD sources of infection

During the 2020 survey year, a total of 443 infected plants were recorded across all fields. Of these, 96.84% (429/443) were infected by cuttings and 3.16% (14/443) by whiteflies. In 2022, a total of 127 infected plants were assessed. Of these, 90.55% (115) were infected cuttings and 9.45% (12) were infected by whiteflies.

### Detection and distribution of cassava mosaic begomoviruses in the surveyed regions

**Fig. 3:**
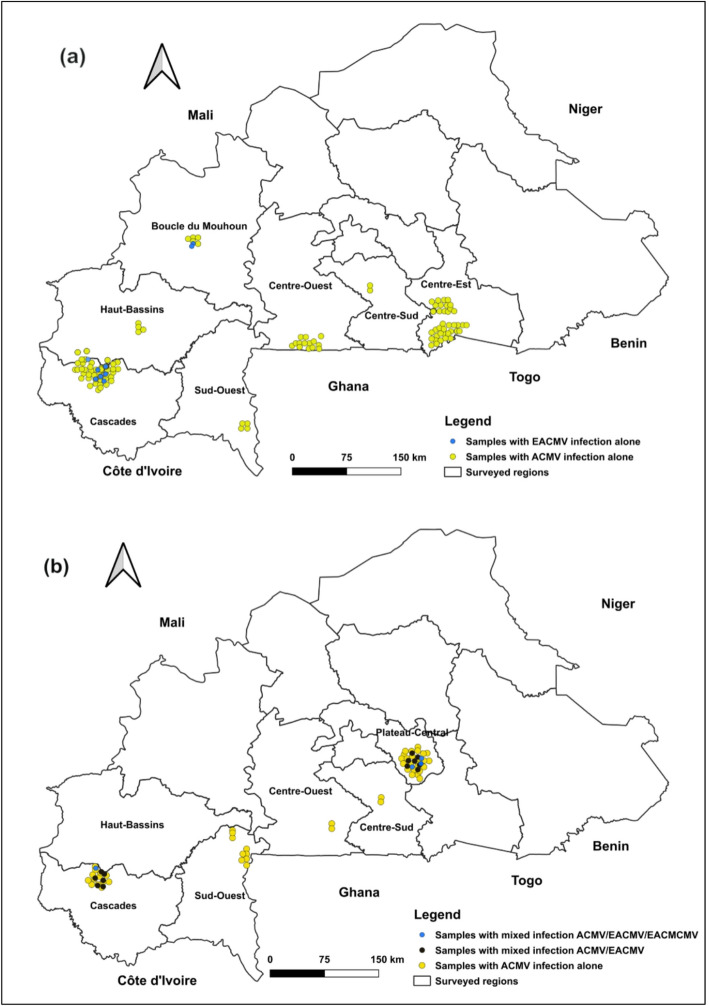
Cassava mosaic begomoviruses species distribution in surveyed regions. (**a**) 2020 survey year. (**b**) 2022 survey year

Based on primers specificity, two CMBs including the ACMV and EACMV virus types, were detected in infected leaf samples during the 2020 survey, and three CMBs including ACMV, EACMV and EACMCMV were detected in the 2023 survey (Fig. 3). PCR analyses were performed on 619 cassava leaves from the 2020 survey, of which 375 were asymptomatic and 244 were symptomatic, and on 350 leaves from the 2022 survey, of which 271 were asymptomatic and 79 were symptomatic (Table 3). During the 2020 survey, the percentage of CMBs detected in symptomatic leaf samples was 97.54% (238/244), while 2.46% (6/244) remained negative to all the primers used. Of the leaves infected with CMBs, the rate of ACMV-like infection alone was 93.85% (229/244). However, the rate of EACMV-like infection alone was 3.69% (9/244). All EACMV-like infections were found in the Boucle du Mouhoun and Cascades regions (Fig. 3a). For the 2022 survey, the total of CMBs detection was 97.47% (77/79) and 2.53% (2/79) remained symptomatic but negative for all the primers. Among the CMB infected leave, 75.95% (60/79) were ACMV-like infection, 16.46% (13/79) were mixed infection including ACMV-like and EACMV-like, and 5.06% (4/79) were mixed infection associating ACMV-like, EACMV-like and EACMCMV. All mixed infections were found in the Cascade and Plateau-Central regions (Fig. 3b).

**Table 3:** Cassava mosaic begomoviruses detection within collected leave samples

Survey year	Tested samples	Infected by CMD	ACMV-like alone (%)	EACMV-like alone (%)	Mixed infection^(a)^ (%)	Mixed infection^(b)^ (%)	Symptomatic negative (%)
2020	619	244	93.85	3.69	0	0	2.46
2022	350	79	75.95	0	16.46	5.06	2.53

ACMV-like alone : samples on which ACMV was detected

EACMV-like alone : samples on which EACMV was detected

Mixed infection (a) : samples on which ACMV and EACMV were detected

Mixed infection (b) : samples on which ACMV, EACMV and EACMCMV were detected

## Discussion

Effective management of plant virus diseases such as CMD requires an efficient method of epidemiological data collection and laboratory diagnosis. Thus, classical surveillance has remained the most widely used method. However, this method has significant limitations that make it difficult to address early management of outbreaks [21]⁠. The aim of this study was to provide recent epidemiological data on CMD in Burkina Faso, while highlighting the limitation of traditional surveillance.

The current study showed that the incidence of CMD in cassava fields was low compared to the incidence of 11.3% and 18.9% found in the 2016 and 2017 surveys by Soro et al. [8], respectively⁠. The incidence found in this study was relatively low compared to the incidence reported in the neighboring countries such as Ivory Coast [22]⁠, Benin [23]⁠, and Ghana [24]⁠. This finding highlights the recent intensive production of cassava in Burkina Faso. In the 2020 survey, we found that the incidence was high in the Centre-Ouest region (23.13%). A similar result was reported by Soro et al. [8]⁠, in the same region during the previous surveys. However, in the 2022 survey, an incidence of 0.66% was recorded in the Centre-Ouest region. This result indicates a decrease in incidence in this region. The reduction in incidence could be due to the application of the CMD management advice given to farmers during 2020 survey. This advice included the adoption of improved varieties and good cultural practices [8]. This result showed that raising farmers’ awareness of how the CMD spreads could help with its management. Also, Burkina Faso experiences high temperature which can up to 40 °C from March to May. This could reduce the incidence of disease because thermotherapy at 40 °C can reduce the viral load in CMD-infected cuttings [25]. Nevertheless, the incidence was high in the Plateau-Central region (32.38%) during 2022 survey. The high incidence could be due to the fact that this region was surveyed for the first time in our study. This result shows that Plateau-Central is a new outbreak of the CMD epidemic. In view of this, it would be necessary to educate cassava farmers in this region about CMD.

In the present study, infection by cuttings was found to be the main source of CMD spread compared to whitefly transmission. This spread could be due to the inability of some cassava farmers to select healthy cuttings when renewing fields. The same result has been observed in previous studies, where the authors found that the spread of infection by cuttings was due to farmers’ ignorance [8, 23, 26]⁠. Therefore, it would be necessary to strengthen the capacity of cassava farmers to select healthy cuttings as planting material.

The low abundance of the whitefly population observed here, reflects its low involvement in the spread of CMD. Similar results have been reported in Benin [23]⁠, Ghana [24]⁠, and Ivory Coast [22]⁠. However, they could be hosted by other plants on which the achieve their life cycle as previously reported in Nigeria [26]⁠.

The current work showed that single ACMV infection was the most predominant in symptomatic leaf samples in all regions surveyed in both survey years. This phenomenon is known to be the most common in all cassava production in West Africa [8, 19, 22, 26, 27]. In addition, the high prevalence of ACMV infection could be related to the high prevalence of moderate symptoms, which are associated with score severity 2. As reported by Pita et al. [17], very severe symptoms in CMD are mostly due to mixed infection. Similarly, we found that EACMV infection was relatively low in all surveyed fields in both survey years. This is not surprising as previous studies have reported that EACMV is more prevalent in East Africa compared to West Africa [15, 17]. The rate of mixed ACMV/EACMV or ACMV/EACMV/EACMCMV infections was low in survey year 2022. Soro et al. [8] found similar results. In addition, mixed infections involving ACMV/EACMV or ACMV/EACMV/EACMCMV were most common in West Africa [19, 22, 26]. This phenomenon may be due to the exchange of cuttings between farmers because of the porous borders in Africa [26].

This study showed that classical epidemiology helps to manage plant virus diseases, but faces significant constraints that make it difficult to address early management. These constraints include the need for financial resources and the time required for field and laboratory activities [21]. It is evident that our study required significant financial resources to cover all the regions surveyed in both survey years. The availability of financial resources is a key element for effective management of plant virus diseases [28]. In most studies based on classical surveillance, plant disease monitoring is carried out by a research team with limited resources and a small staff for regional or national surveillance. Due to these resource and staff limitations in developing countries, researchers are sometimes unable to cover all areas of interest for disease assessment [29]. In addition, PCR diagnosis requires the availability of laboratory infrastructure. However, setting up fully equipped laboratory infrastructures and ensuring the availability of reagents for their operation is extremely costly. As reported by Baksh et al. [21], the high cost of laboratory diagnosis is one of the main limitations of classical surveillance. Traditional surveillance methods rely on visual inspection of symptoms to assess disease. However, this method is time consuming in large scale surveillance. It was therefore not surprising that our study spent so much time on field activities alone. In this regard, recent studies comparing field diagnostic methods have reported that the assessment of plant virus disease symptoms based on visual observation is not only laborious but also time-consuming [30, 31]. Similarly, although laboratories are available, they are not practical for field diagnosis. PCR diagnosis involves the extraction and quantification of DNA samples from collected leaf samples for virus detection. As observed in the current study, it was clear that carrying out the diagnosis of 619 or 350 samples could be time consuming to make the diagnostic results available. Because of this time-consuming nature, PCR methods are considered a limitation of classical surveillance [32]. Similarly, in our study, some leave-behind samples with symptoms consistent with CMD remained negative for PCR detection. This result is similar to most studies using PCR methods [8, 19, 22, 33]. PCR methods are sometimes limited, due to primers specificity for virus detection accurately, which can lead to negative results [34]. This phenomenon, attributed to the evolutionary dynamics and genetic variability of viruses, has been reported as one of the limitations of PCR methods [34].

The limitations highlighted in our study show that classical surveillance methods do not allow the anticipation of alerts for early management of plant diseases. However, early detection is a crucial step in anticipating effective management of plant diseases. In view of this, the implementation of a surveillance approach such as participatory surveillance, which includes and involves farmers, extension agents, breeders, phytosanitary authorities and researchers as actors in the disease surveillance chain, could help to overcome classical surveillance constraints [35, 36]. Widely developed and applied in the field of human and animal health, participatory surveillance has provided satisfactory results in the management of Covid 19 in Brazil [37], Ebola hemorrhagic fever in the Republic of Guinea [38], and zoonosis in Kenya [39] and Ethiopia [40]. However, its application in plant health field as a surveillance approach has received little attention. To this end, the use of artificial intelligence tools for early diagnosis of plant diseases has become essential [12, 41–44]. With these tools, participatory surveillance approaches are increasingly showing promising results for early detection of plant diseases for early management [45, 46]. In addition, Oxford nanopore sequencing device MinION offer advantages to overcome the limitations of PCR methods for in-field diagnosis [47, 48].

## Conclusion

Our study allowed us to update the epidemiological data of cassava mosaic disease in Burkina Faso, based on classical surveillance methods. Globally, we found that the incidence of CMD decreased progressively from 2017 to 2022. The Plateau-Central region was identified as an emerging CMD epidemic. Transmission by cuttings remains the main mode of CMD spread. ACMV infection was more prevalent than EACMV. We found several re-emerging mixed infections involving ACMV/EACMV and ACMV/EACMV/EACMCMV. However, the main constraints observed in the implementation of classical surveillance are its high cost and time consuming due to field and laboratory activities. Nevertheless, to overcome these constraints, implementation of innovative methods including participatory surveillance with artificial intelligence tools and Oxford nanopore portable sequencing advisory could be an alternative for early management of plant diseases and especially CMD. This implementation throughout farmer’s, extension agents, breeders, phytosanitary authorities and researchers as actors could help to real-time surveillance which allow early management of plant diseases.

## Supplementary Information

Below is the link to the electronic supplementary material.

Supplementary Material 1

## Data Availability

Datasets generated during the current study are available from the corresponding author upon reasonable request.
