# Supplementary material for: Cassava mosaic disease in Burkina Faso: epidemiological aspects and disease management perspectives
Source: Discov Agric. 2025 Oct 7;3(1):197. doi: 10.1007/s44279-025-00386-2 (PMC12504380; doi:10.1007/s44279-025-00386-2)
Supplement: Supplementary file 1 — Supplementary Material 1 [file 44279_2025_386_MOESM1_ESM.pptx]

## Slide 1
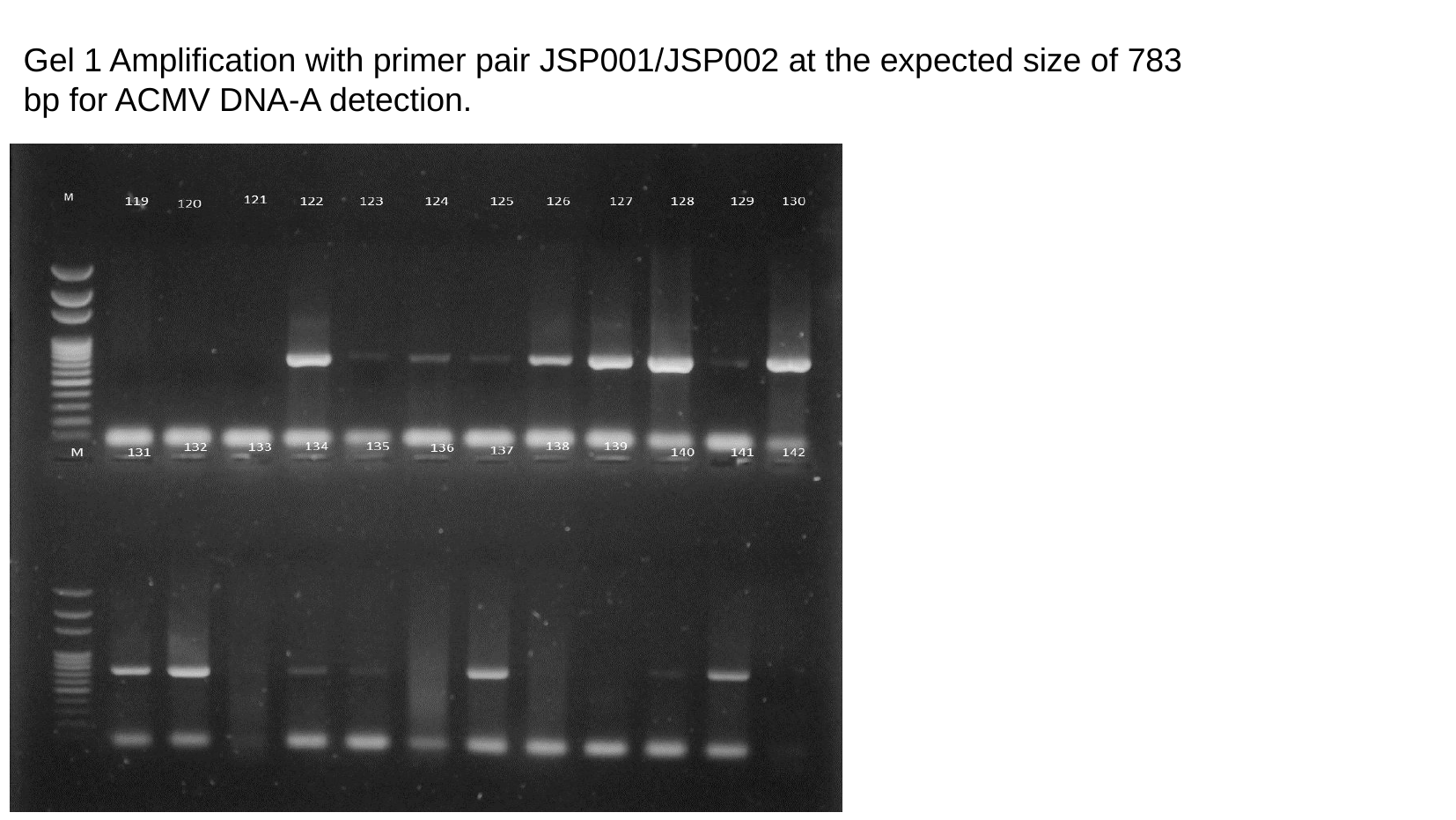

Gel 1 Amplification with primer pair JSP001/JSP002 at the expected size of 783 bp for ACMV DNA-A detection.

## Slide 2
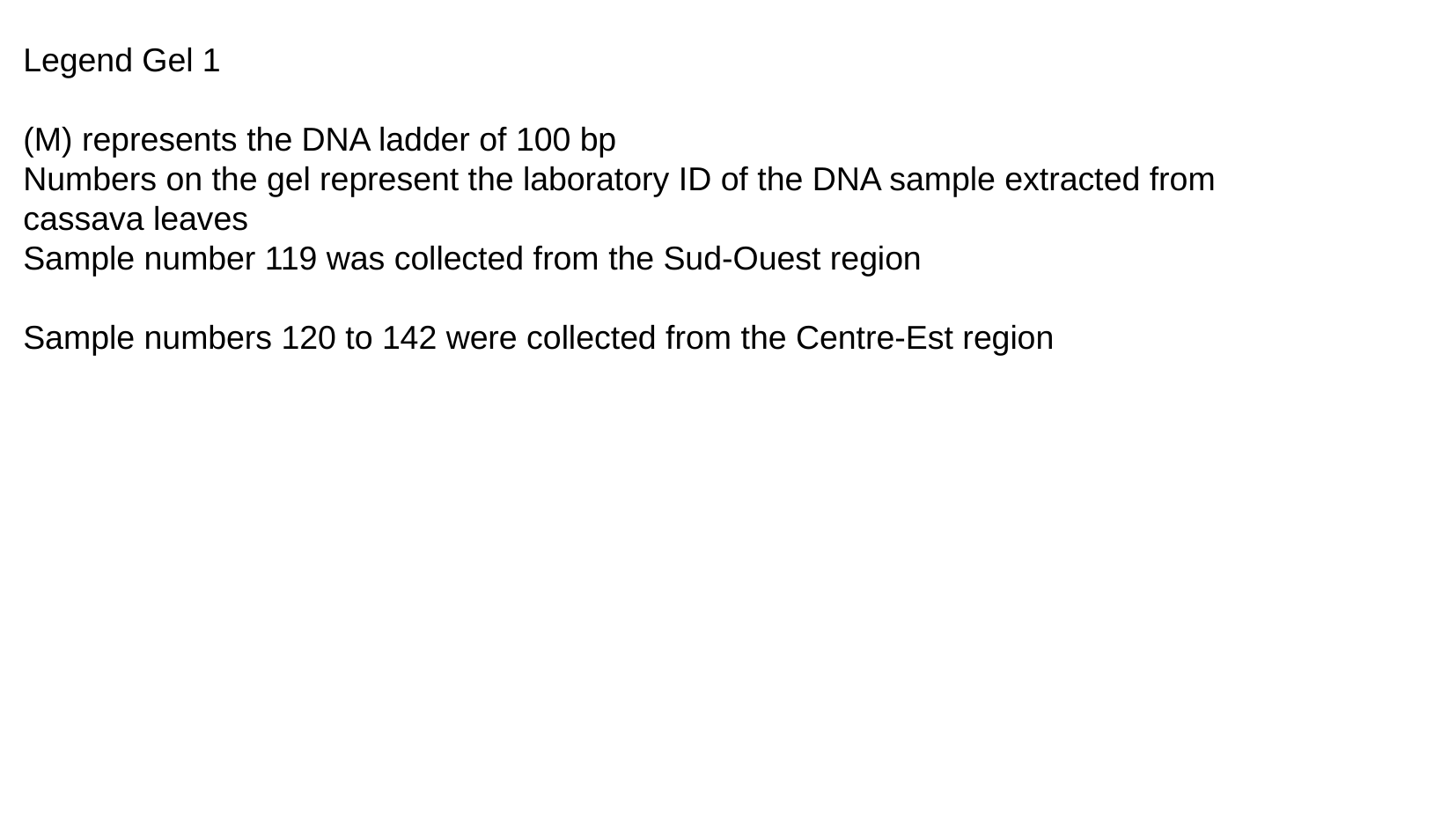

Legend Gel 1
(M) represents the DNA ladder of 100 bp
Numbers on the gel represent the laboratory ID of the DNA sample extracted from cassava leaves
Sample number 119 was collected from the Sud-Ouest region
Sample numbers 120 to 142 were collected from the Centre-Est region

## Slide 3
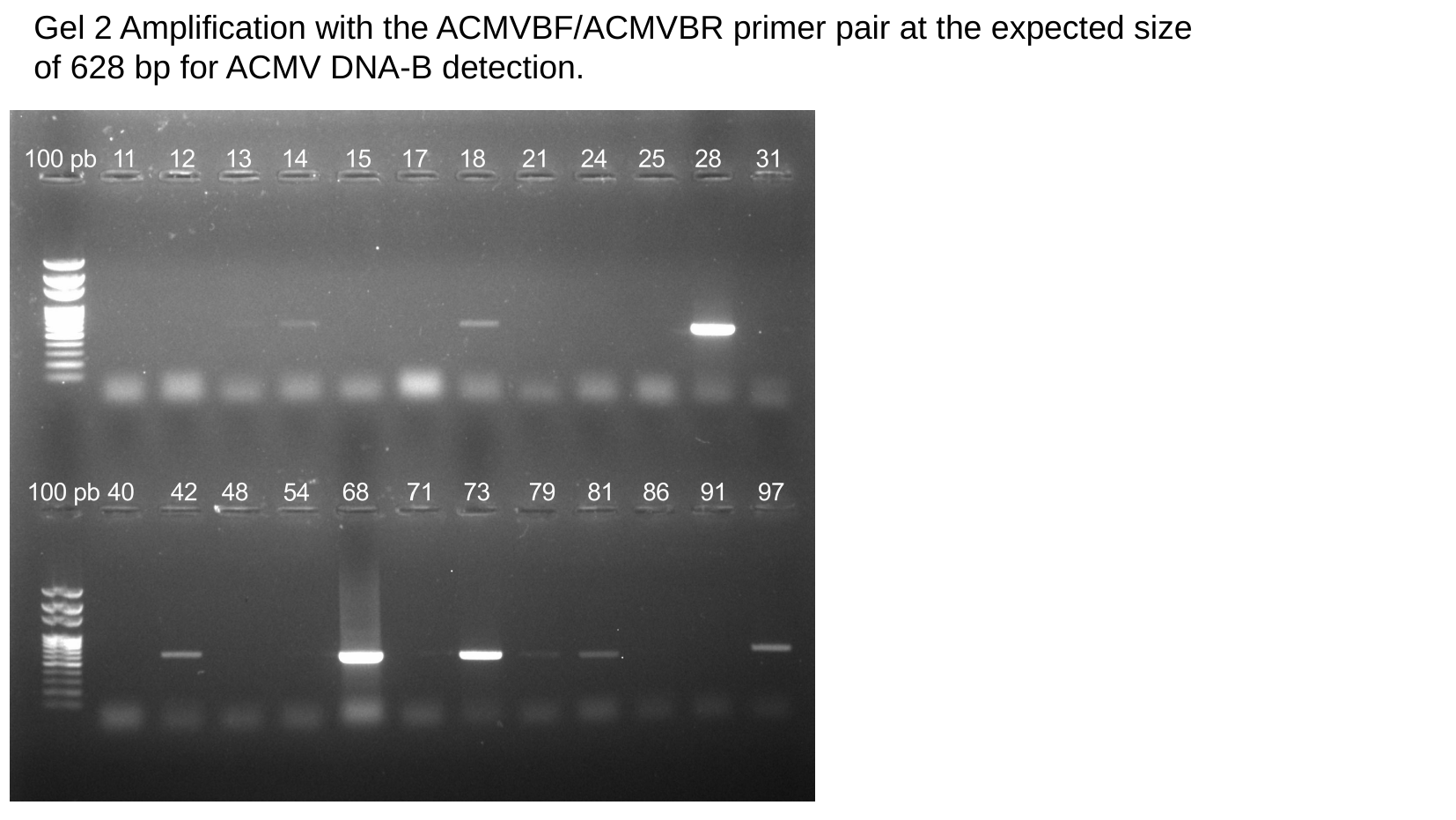

Gel 2 Amplification with the ACMVBF/ACMVBR primer pair at the expected size of 628 bp for ACMV DNA-B detection.

## Slide 4
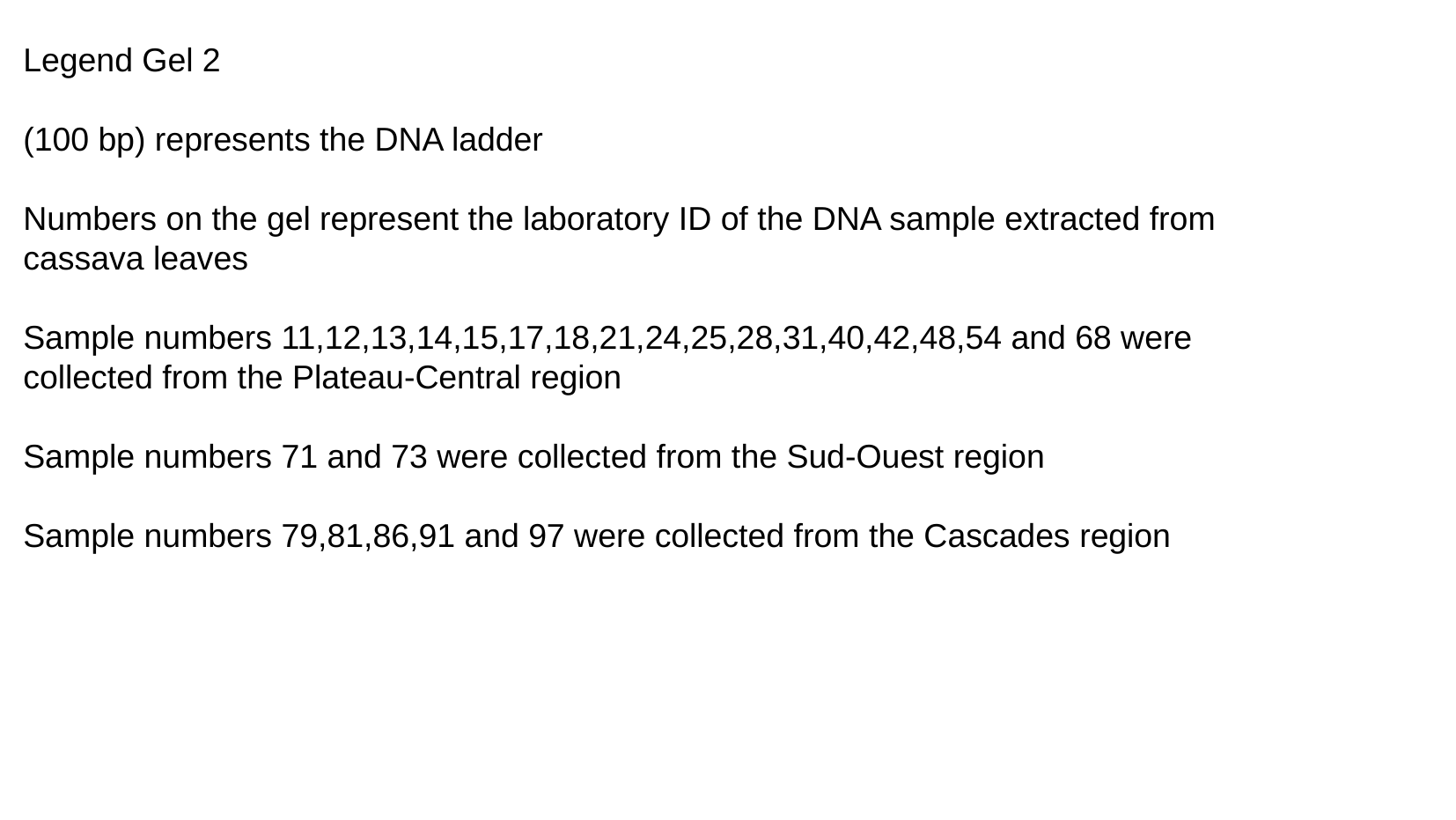

Legend Gel 2
(100 bp) represents the DNA ladder
Numbers on the gel represent the laboratory ID of the DNA sample extracted from cassava leaves
Sample numbers 11,12,13,14,15,17,18,21,24,25,28,31,40,42,48,54 and 68 were collected from the Plateau-Central region
Sample numbers 71 and 73 were collected from the Sud-Ouest region
Sample numbers 79,81,86,91 and 97 were collected from the Cascades region

## Slide 5
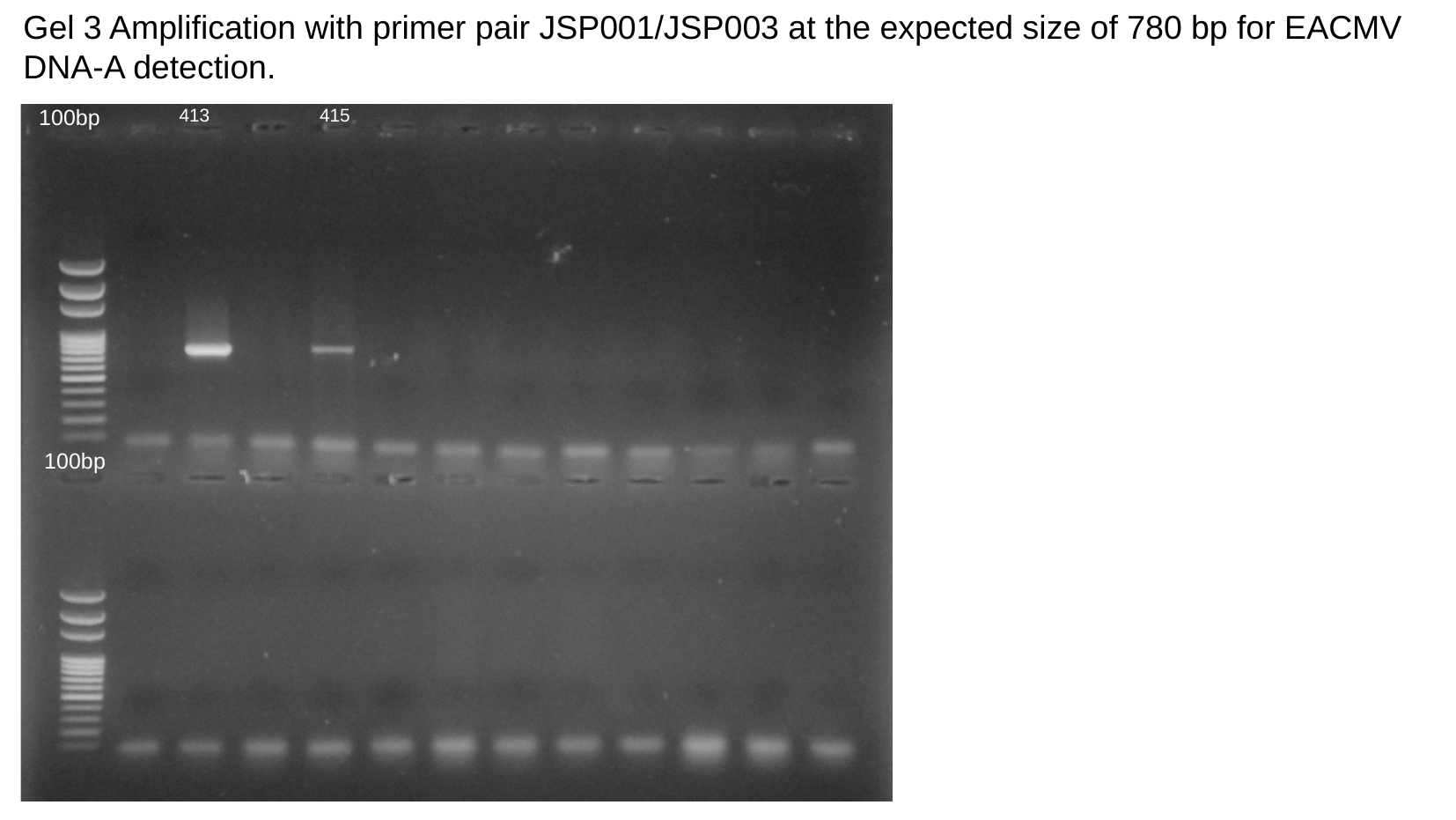

Gel 3 Amplification with primer pair JSP001/JSP003 at the expected size of 780 bp for EACMV DNA-A detection.
415
100bp
413
100bp

## Slide 6
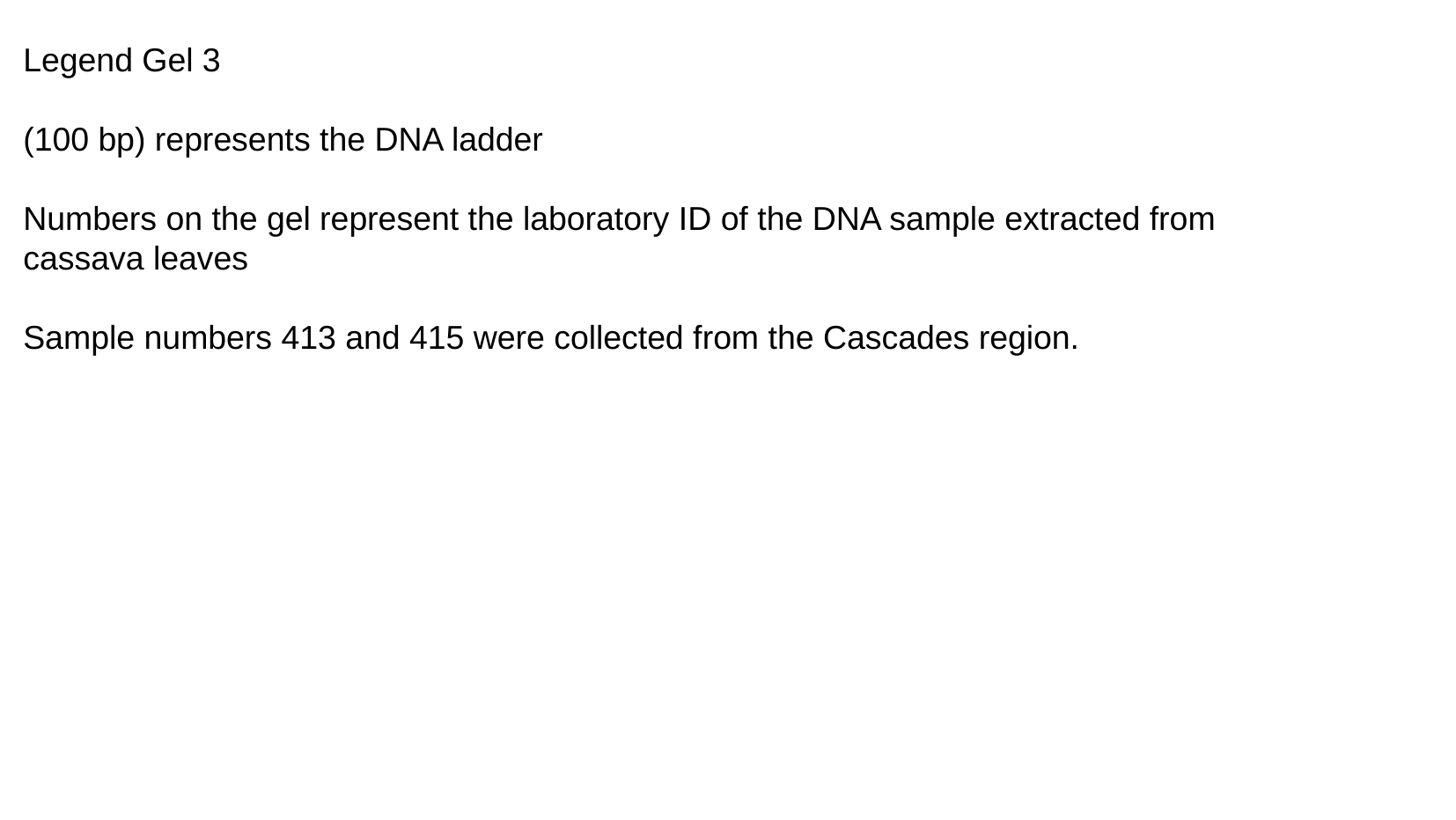

Legend Gel 3
(100 bp) represents the DNA ladder
Numbers on the gel represent the laboratory ID of the DNA sample extracted from cassava leaves
Sample numbers 413 and 415 were collected from the Cascades region.

## Slide 7
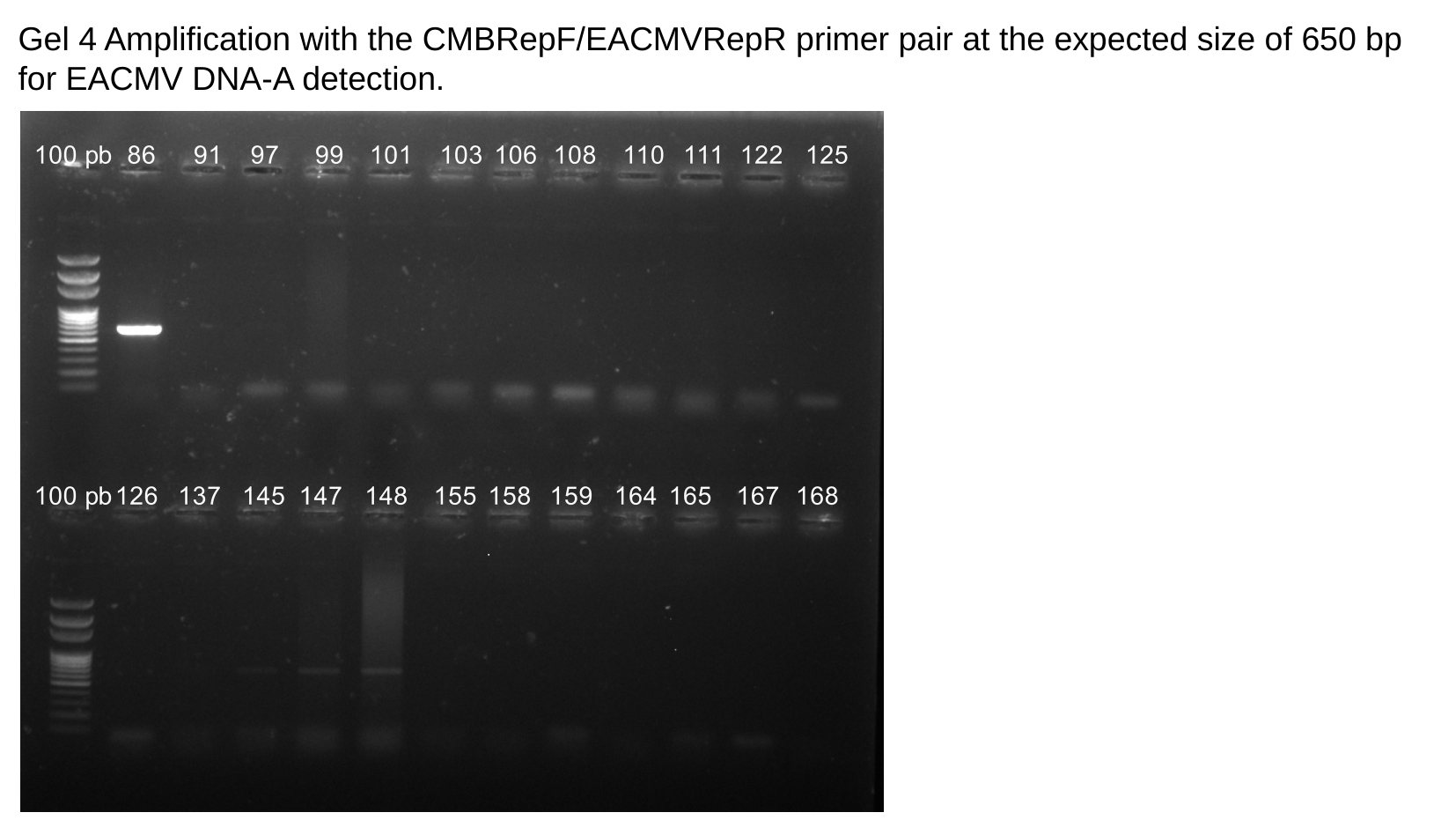

Gel 4 Amplification with the CMBRepF/EACMVRepR primer pair at the expected size of 650 bp for EACMV DNA-A detection.

## Slide 8
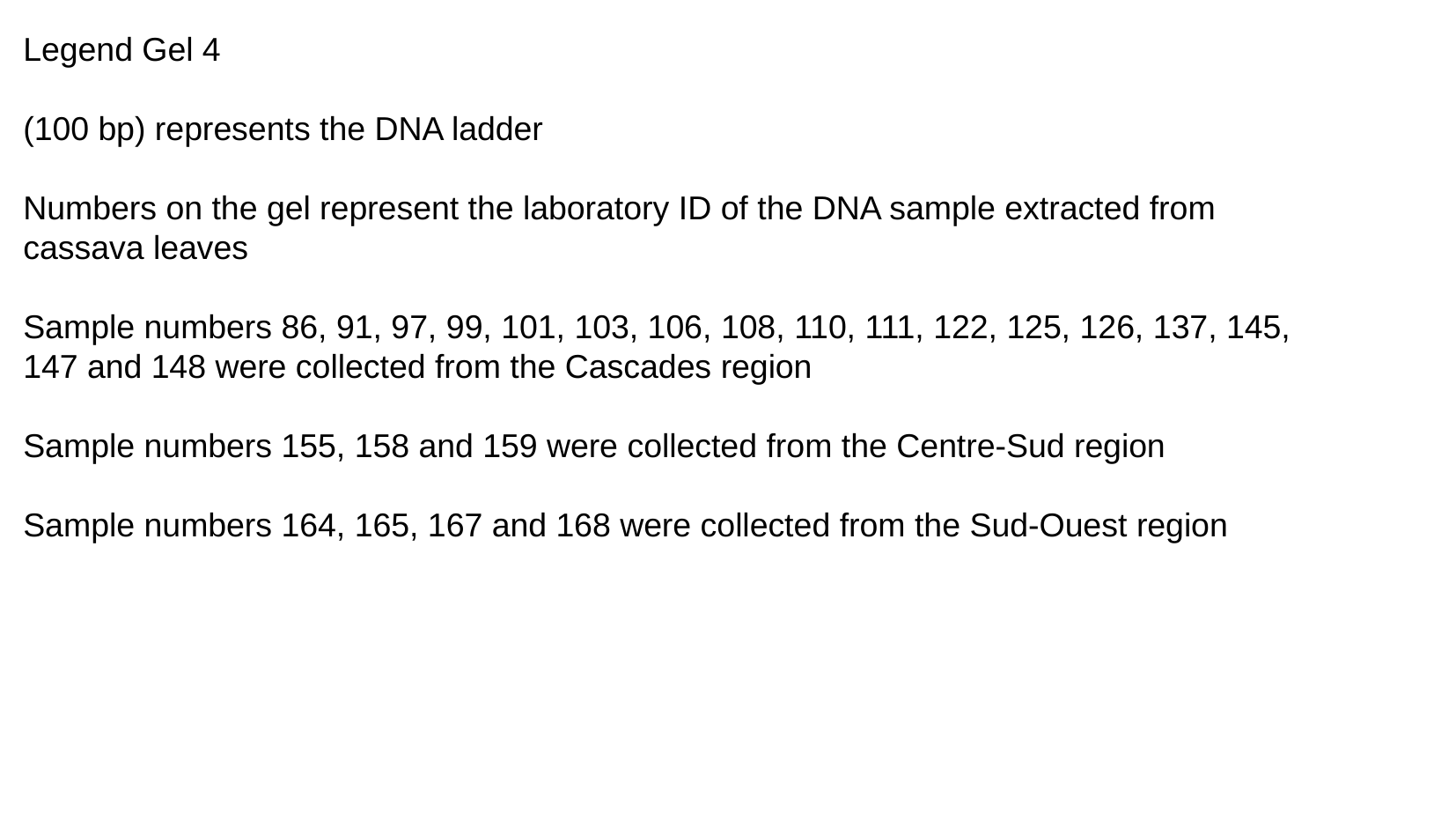

Legend Gel 4
(100 bp) represents the DNA ladder
Numbers on the gel represent the laboratory ID of the DNA sample extracted from cassava leaves
Sample numbers 86, 91, 97, 99, 101, 103, 106, 108, 110, 111, 122, 125, 126, 137, 145, 147 and 148 were collected from the Cascades region
Sample numbers 155, 158 and 159 were collected from the Centre-Sud region
Sample numbers 164, 165, 167 and 168 were collected from the Sud-Ouest region

## Slide 9
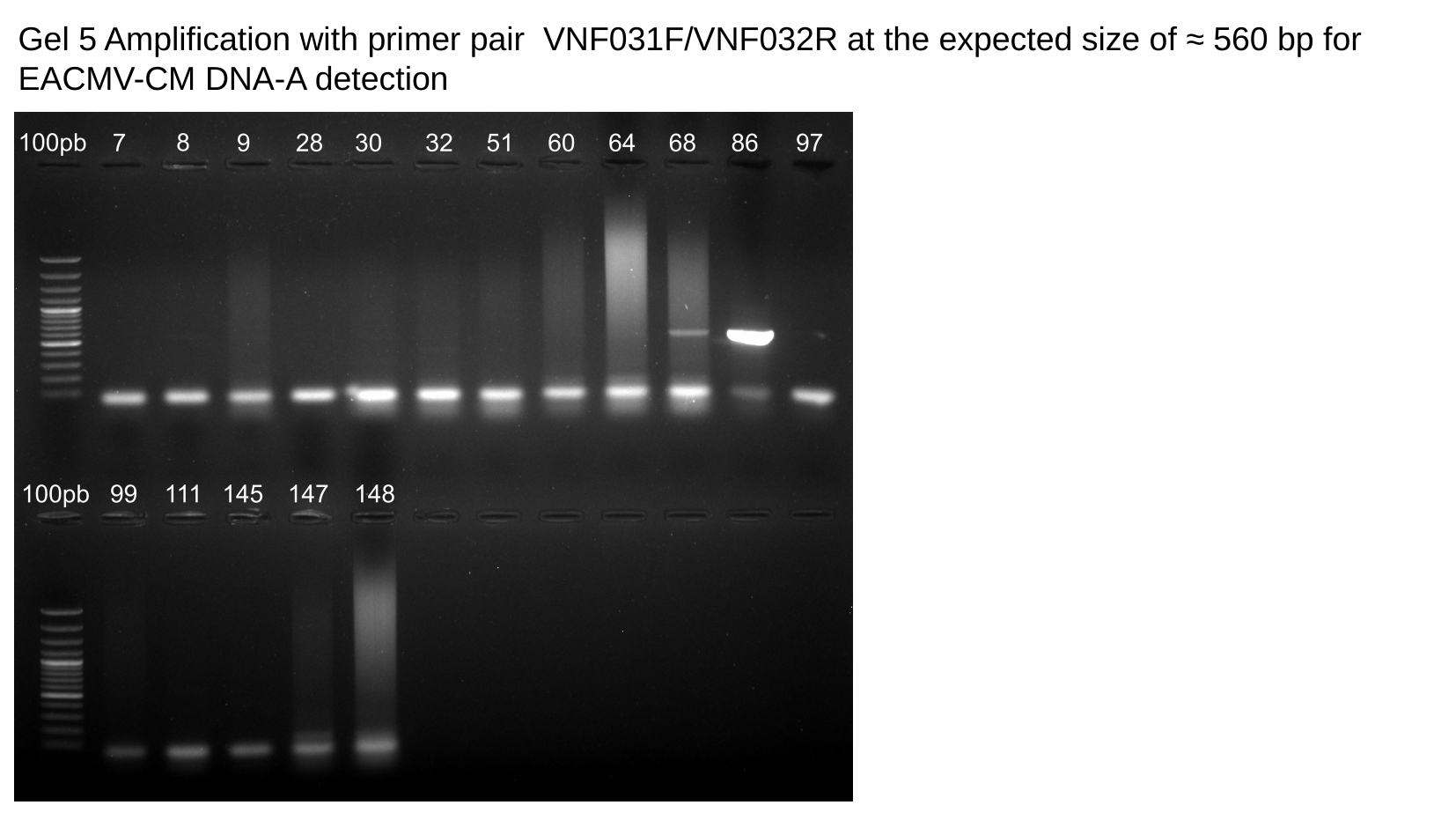

Gel 5 Amplification with primer pair VNF031F/VNF032R at the expected size of ≈ 560 bp for EACMV-CM DNA-A detection

## Slide 10
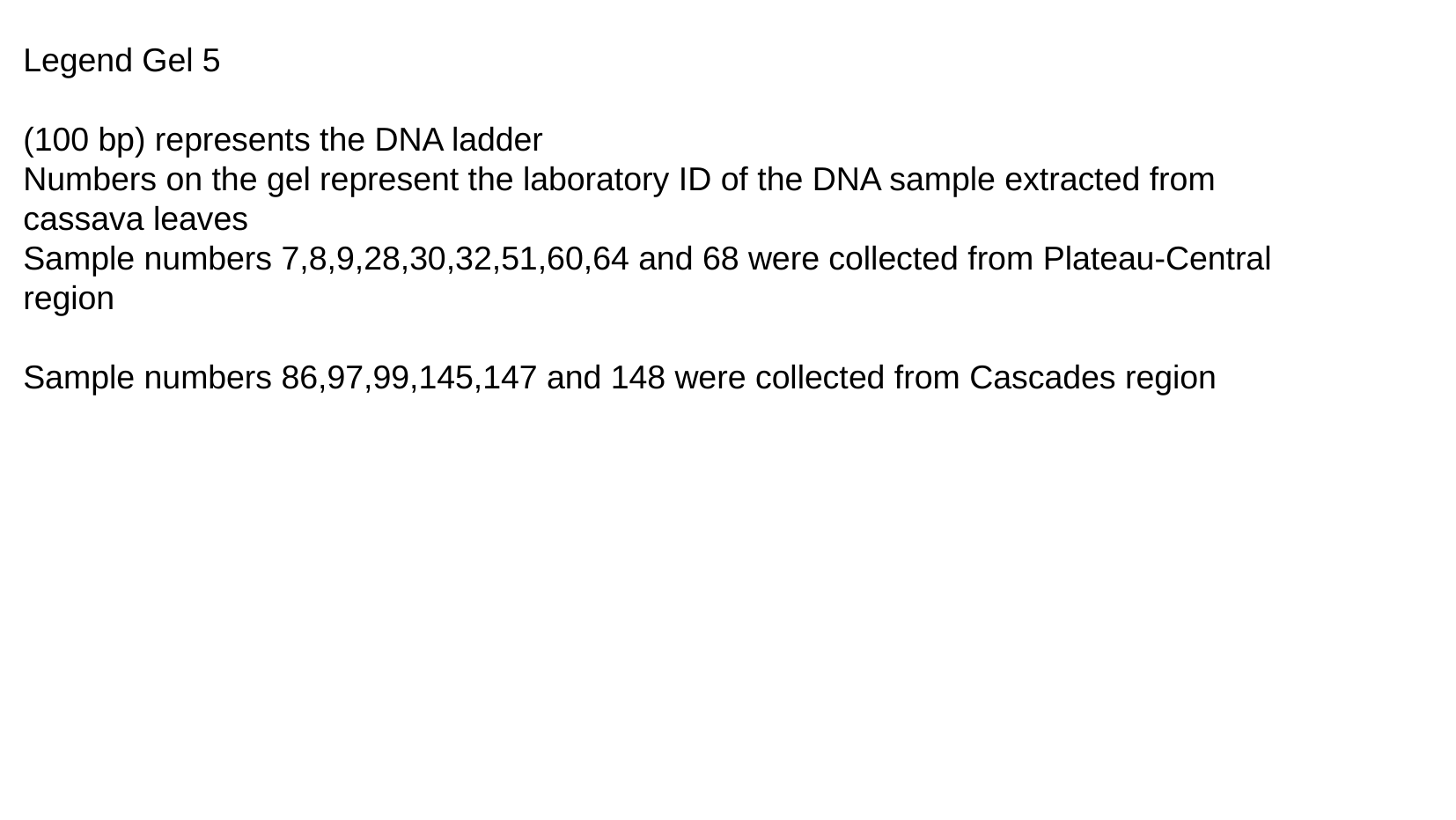

Legend Gel 5
(100 bp) represents the DNA ladder
Numbers on the gel represent the laboratory ID of the DNA sample extracted from cassava leaves
Sample numbers 7,8,9,28,30,32,51,60,64 and 68 were collected from Plateau-Central region
Sample numbers 86,97,99,145,147 and 148 were collected from Cascades region

## Slide 11
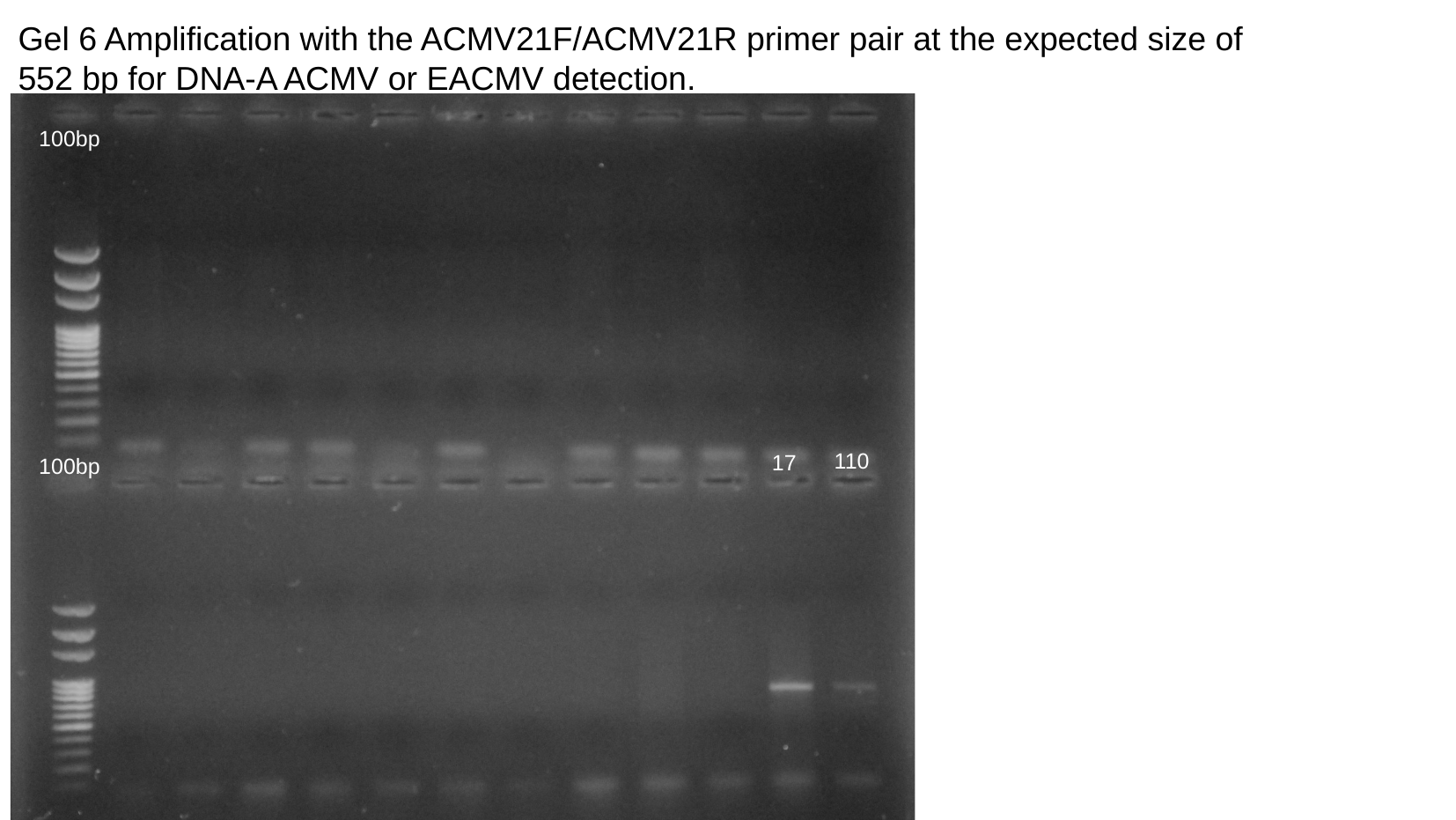

Gel 6 Amplification with the ACMV21F/ACMV21R primer pair at the expected size of 552 bp for DNA-A ACMV or EACMV detection.
100bp
110
17
100bp

## Slide 12
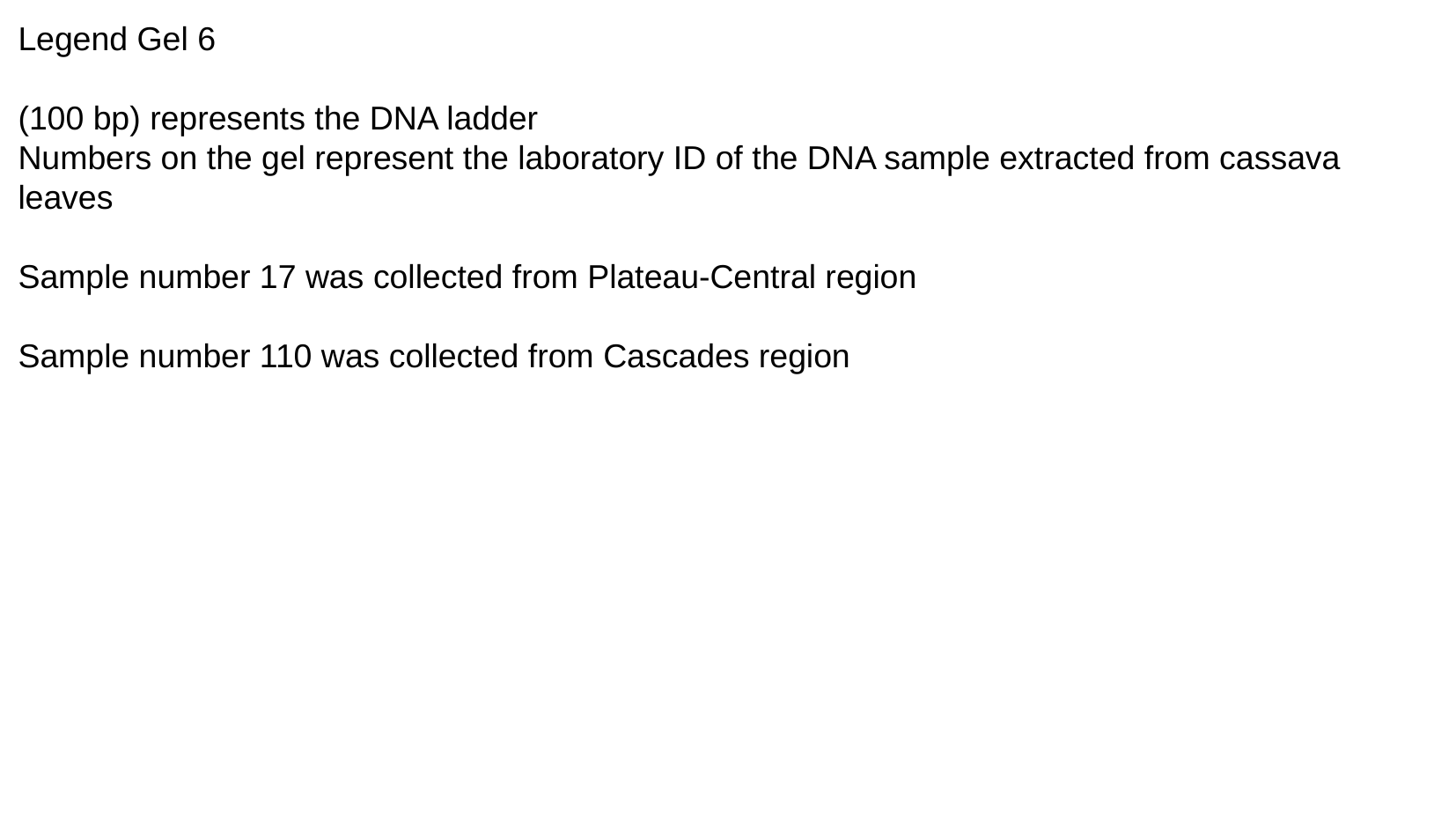

Legend Gel 6
(100 bp) represents the DNA ladder
Numbers on the gel represent the laboratory ID of the DNA sample extracted from cassava leaves
Sample number 17 was collected from Plateau-Central region
Sample number 110 was collected from Cascades region

## Slide 13
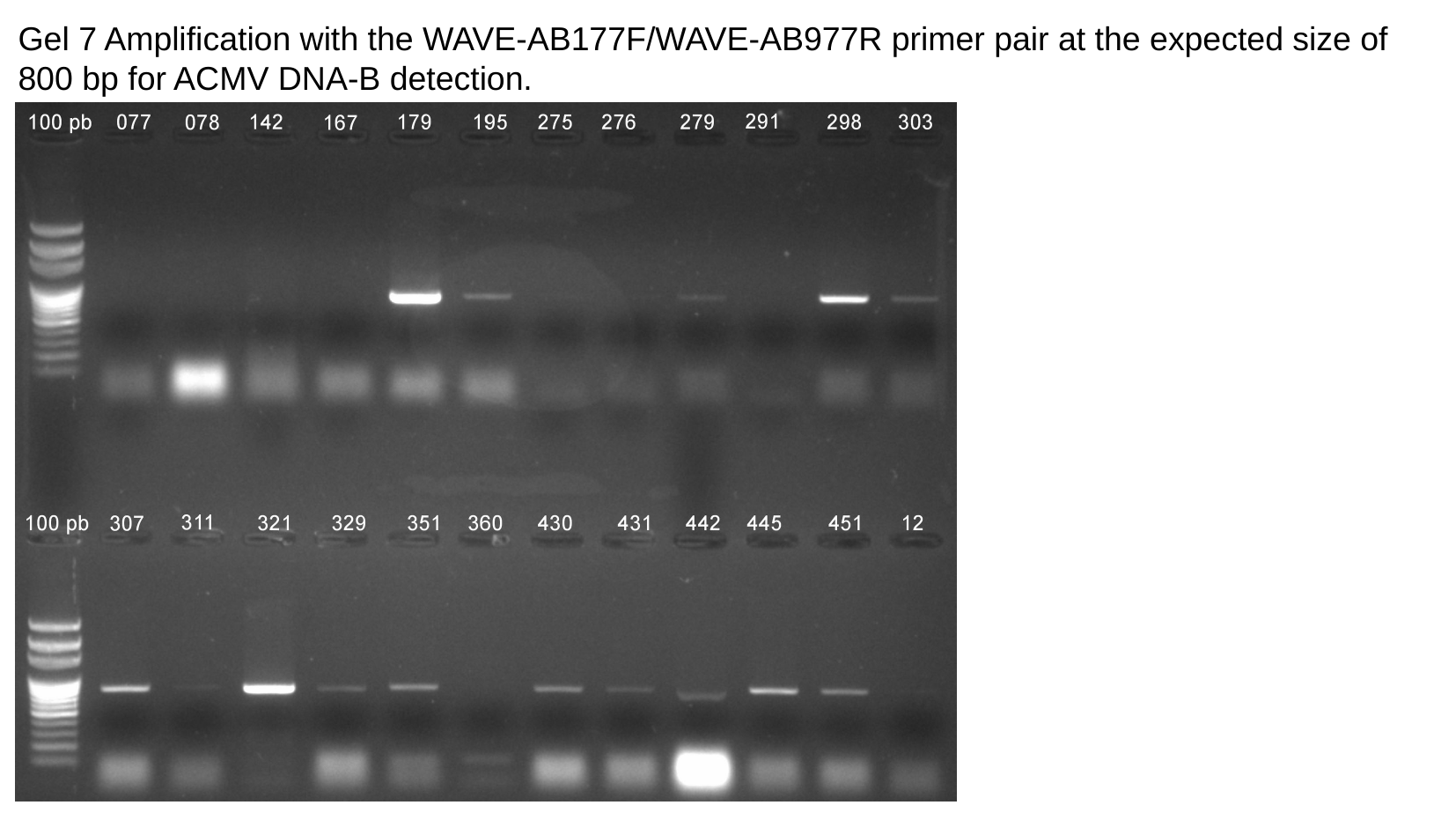

Gel 7 Amplification with the WAVE-AB177F/WAVE-AB977R primer pair at the expected size of 800 bp for ACMV DNA-B detection.

## Slide 14
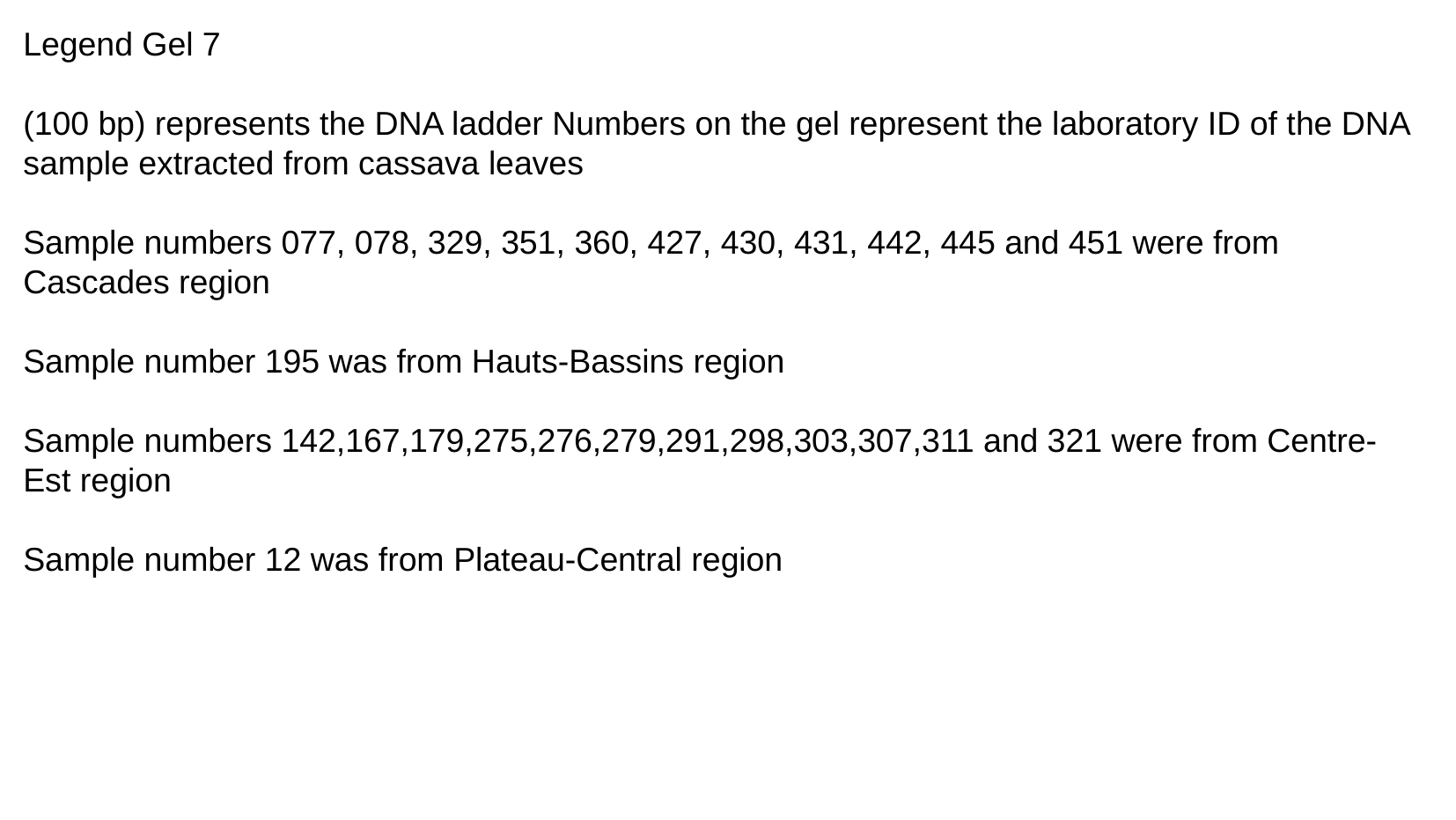

Legend Gel 7
(100 bp) represents the DNA ladder Numbers on the gel represent the laboratory ID of the DNA sample extracted from cassava leaves
Sample numbers 077, 078, 329, 351, 360, 427, 430, 431, 442, 445 and 451 were from Cascades region
Sample number 195 was from Hauts-Bassins region
Sample numbers 142,167,179,275,276,279,291,298,303,307,311 and 321 were from Centre-Est region
Sample number 12 was from Plateau-Central region
